# Regional impairment of deep gray matter perfusion in neonates with congenital heart disease revealed by arterial spin labeling MRI

**DOI:** 10.3389/fnhum.2022.803006

**Published:** 2022-09-02

**Authors:** Yan Sun, Yujie Liu, Wenwen Yu, Yumin Zhong

**Affiliations:** ^1^Department of Radiology, Shanghai Children's Medical Center, School of Medicine, Shanghai Jiao Tong University, Shanghai, China; ^2^Department of Cardiovascular and Thoracic Surgery, Shanghai Children's Medical Center, School of Medicine, Shanghai Jiao Tong University, Shanghai, China; ^3^Institute of Science and Technology for Brain-Inspired Intelligence, Fudan University, Shanghai, China

**Keywords:** congenital heart disease, neonates, arterial spin labeling, MRI, perfusion

## Abstract

The present study examined deep gray matter perfusion in neonates with congenital heart disease (CHD) with arterial spin labeling magnetic resonance imaging preoperatively. We found that neonates with cyanotic CHD showed lower right thalamus compared with controls and lower right basal ganglia perfusion compared with acyanotic CHD. When the CHD group was assessed as a whole, it showed slightly decreased left thalamus perfusion compared with controls. The results suggest that cardiac physiology plays a crucial part in changes in regional cerebral perfusion. Perfusion with arterial spin labeling may be a useful marker of high risk for impaired cerebral blood flow auto-regulation and cerebral hypoperfusion in neonates with CHD.

## Introduction

Congenital heart disease (CHD) is among the most common birth defects, accounting for one-third of all congenital malformations (Leonetti et al., 2019). Potent and healthy cardiovascular function play an important role in maintaining neurodevelopment. These defects apparently cause abnormal circulation that may further lead to abnormal regulation of cerebral perfusion (Donofrio et al., 2003). Despite significant improvements in diagnostic strategies, surgical management, and perioperative care, children with CHD are rather vulnerable to heightened risks of brain injury and neurodevelopmental disorders affecting gross and fine motor skills, intellectual function, memory, executive function, speech, and language, all of which could continue into adulthood (Jakab et al., 2019). Therefore, it is important to identify potential risk factors for cerebral injury and neurodevelopmental outcomes in this population. However, it is most likely multifactorial and not fully understood up to date (Marelli et al., 2016).

Accumulating evidence obtained from multi-modal magnetic resonance imaging (MRI) techniques indicates that fetuses and neonates with CHD often exhibit altered cortical morphology, including reduced gray and white matter volume, white matter microstructural immaturity, and abnormal cortical folding (Miller et al., 2007; Limperopoulos et al., 2010; Clouchoux et al., 2013; Von Rhein et al., 2015). Furthermore, decreased brain maturation has been reported in more specific CHD types, especially in complex CHD, such as hypoplastic left heart syndrome (HLHS) and D-transposition of the great arteries (D-TGA). Jørgensen et al. (2018) found that patients with D-TGA have reduced cerebral growth rates. Moreover, using near-infrared spectroscopy, a study has shown impaired cerebral autoregulation in CHD newborns during the first few hours after birth (Votava-Smith et al., 2017). Collectively, these findings suggest that hemodynamic factors may contribute to abnormal neurodevelopment with CHD.

Arterial spin labeling (ASL) MR perfusion offers a noninvasive way to measure cerebral blood flow (CBF) and evaluate regional perfusion differences by using water molecules in arterial blood as an endogenous tracer (Haller et al., 2016). In the recent years, it has proven to be highly suitable for neonates and children, since it can provide absolute quantification of CBF without contrast material administration or radiation and is repeatable (Tortora et al., 2017, 2020; Proisy et al., 2019). It has been used to quantitatively evaluate cerebral perfusion changes in neonatal hypoxic-ischemic encephalopathy and preterm newborns. To date, only two studies have examined cerebral perfusion in CHD newborns with ASL. Licht et al. (2004) demonstrated lower preoperative global CBF in CHD neonates with ASL. Nagaraj et al. (2015) reported that neonates with complex CHD showed disturbed CBF compared to controls by ASL. They demonstrated the importance of abnormal hemodynamic status for altered cerebral perfusion.

CHD can be divided into cyanotic and acyanotic anomalies. In both types, the intracardiac defects or extracardiac great vessel anomalies result in difficulty in pumping blood as efficiently as it should do. The main difference between the two types is that cyanotic CHD causes the mixing of blood in systemic and pulmonary circulation, leading to low levels of oxygen saturation of the blood and cyanosis (Ernstene, 1932). Often, more than one defect is present in cyanotic CHD. Oppositely acyanotic CHD does not mix systemic and pulmonary venous blood or result in low levels of oxygen in the blood. How the different and complicated physiological changes in the blood circulation of cyanotic and acyanotic CHD impact cerebral perfusion remains unclear. Some studies have investigated this. As mentioned earlier, Nagaraj et al. (2015) demonstrated the perfusion of occipital white matter, thalamus, and basal ganglia (BG) in cyanotic CHD was reduced compared with that in acyanotic CHD. Shillingford et al. (2007) found that in newborns with HLHS, the ascending aorta of those with microcephaly is significantly smaller than those without microcephaly, indicating the complex interaction of cerebral hemodynamics in this complex CHD type.

The deep gray matter of the brain plays a crucial role in learning, behavior, cognition, movement, and memory. Its development is fundamental to cerebral networks (Arsalidou et al., 2013). According to systematic reviews, researchers found deep gray matter with smaller volumes in CHD neonates and fetuses (Von Rhein et al., 2015). So, there is a question about how the perfusion in deep gray matter in CHD neonates, especially in cyanotic CHD. The previous studies are limited. Thus, we hypothesized that CHD neonates would present lower CBF in this region than control neonates. Accordingly, we aimed to compare preoperative deep gray matter perfusion obtained with ASL in CHD neonates with healthy controls and explore possible CBF differences between two types of CHD.

## Materials and methods

### Participant

Between 2018 and 2020, newborns with CHD requiring surgical intervention with cardiopulmonary bypass within the first month at our hospital were enrolled in this prospective study. The study was approved by an ethics board. Written informed consent for the use of data for research purposes was signed by the parents of participants before MR imaging. The inclusion criteria included: (1) gestational age > 36 weeks; (2) before cardiac surgery; (3) medically stable for 24 h before imaging. Exclusion criteria included: (1) history of birth asphyxia; (2) preoperative cardiac arrest requiring chest compressions; (3) congenital infection; (4) congenital malformation; and (5) known or suspected genetic abnormalities. Healthy newborn controls were recruited from healthy pregnant volunteers and were screened by ultrasound for no intracranial lesions or congenital heart disease.

### Data acquisition

Neonatal cerebral MRI was performed in natural sleep on a 3 Tesla scanner (Discovery MR750; GE Healthcare) with the use of a 32-channel head coil. We used the feed and bundle method to acquire MR imaging without sedation. The axial 3-dimensional pseudocontinuous ASL sequence was performed with the following parameters: TE, 11.1 ms; TR, 4,562 ms; voxel size, 0.8 × 0.8 × 3 mm^3^, postlabeling delay, 1,025 ms, labeling duration, 1.450 s. The imaging plane was positioned at the base of the pons. Anatomic sequences included sagittal 3D T1 BRAVO (TR, 6.6 ms, TE, 2.5 ms, TI, 800 ms, voxel size, and 1×1×1 mm^3^), and axial T2-fluid attenuated inversion recovery (FLAIR) sequence (TR, 9,000 ms, TE, 120 ms, TI, 2,469 ms, FOV, 200 mm × 200 mm, and slice thickness, 4 mm). Axial T2^*^-weighted susceptibility angiography (TE, 25 ms; TR, 46.9 ms; FOV, 200 × 200 mm; and section thickness, 2 mm) and diffusion-weighted image (DWI, TE, 45 ms; TR, 3,000; FOV, 200 × 200 mm; and section thickness, 2.8 mm) were obtained and assessed for signs of brain injury. Oxygen saturation and heart rate were monitored during the MR scan by a pediatrician. Pulse oximetry, temperature, electrocardiography and respiratory rate were supervised by a doctor from the department of cardiovascular and thoracic surgery with an MR monitoring system during the MR examinations.

### Image post-processing

**Figure 1:**
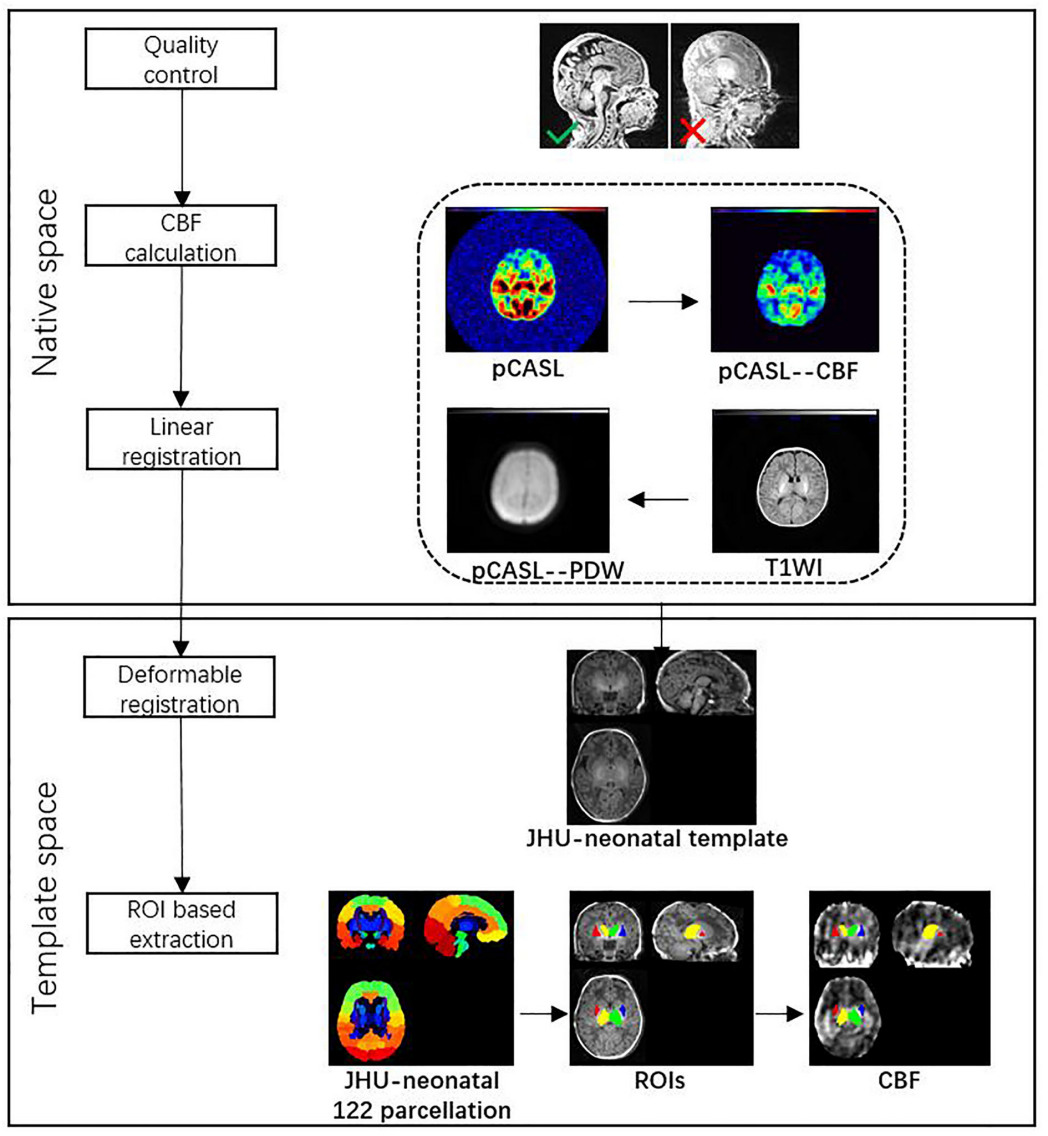
Data analysis pipeline.

**Figure 2:**
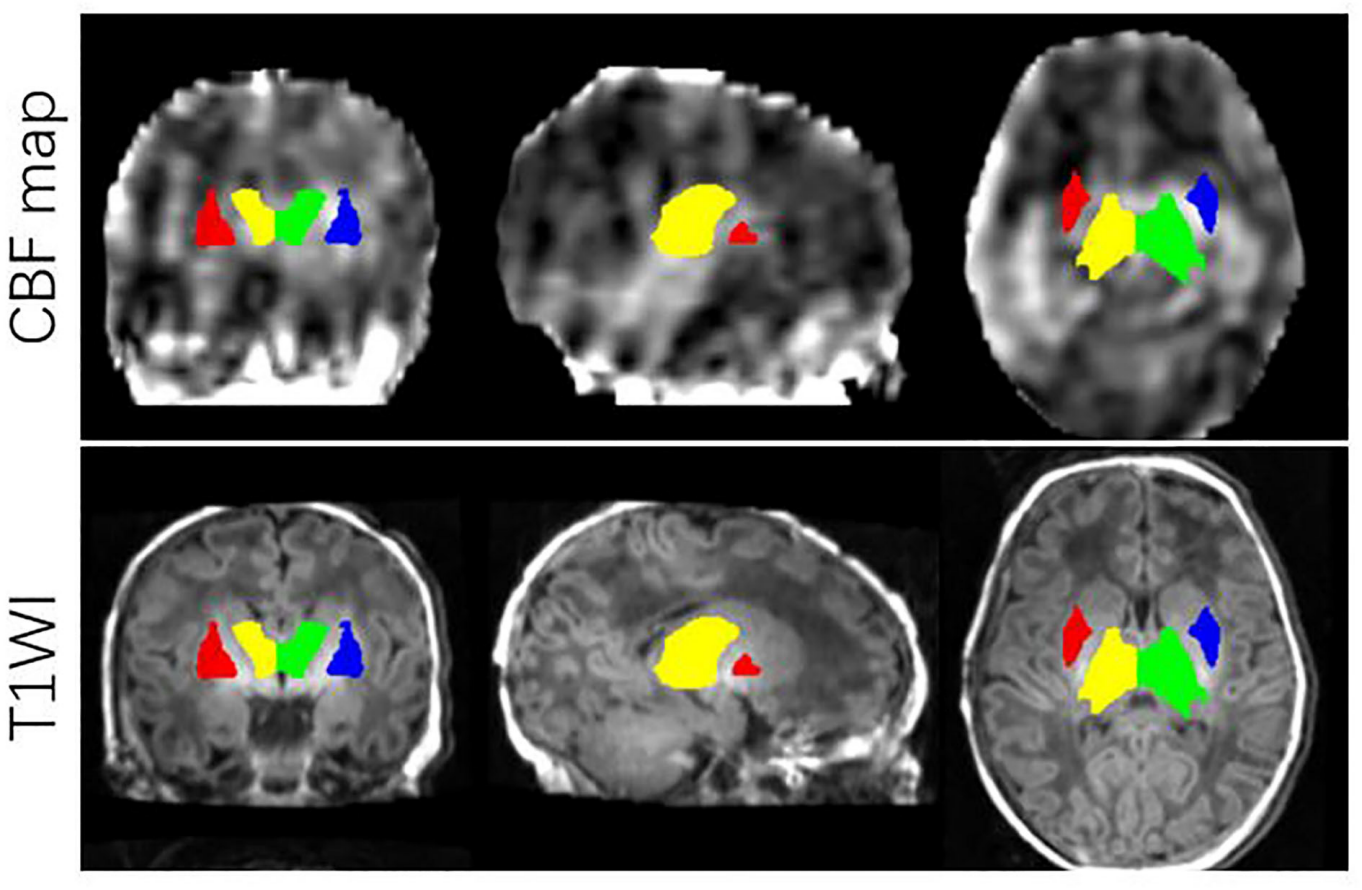
ROIs on JHU-neonatal brain atlas (lower panels) and CBF map (upper panels). ROI locations include right thalamus (yellow), left thalamus (green), right BG (red) and left BG (blue).

All images from subjects were inspected by two pediatric neuroradiologists to exclude the data with artifacts such as motion artifacts and assure reliable quality. The neuroradiologists should be blinded to the clinical information and condition of the CHD patients and controls. The flowchart of data analysis is illustrated in Figure 1. In an initial step, CBF images were acquired with Functool software on an Advantage Windows Workstation (GE Healthcare). The whole brain T1-weighted images of each individual were co-registered rigidly to their own Proton Density (PD) weighted images with the consistent orientation and resolution as CBF images using Statistical Parametric Mapping 8 (SPM 8, http://www.fil.ion.ucl.ac.uk/spm). After registering images within each individual, the T1 weighted volumes were normalized to JHU neonate single subject T1-weighted atlas (Oishi et al., 2011) through affine non-rigid registration using ANTs (Advanced Neuroimaging Tools, http://stnava.github.io/ANTs). Additionally, corresponding CBF maps can be registered into the same Atlas space for subsequent regions of interest (ROI)-based analysis. Based on the importance of the deep gray matter in the brain (Arsalidou et al., 2013; Von Rhein et al., 2015), we finally selected 4 ROIs including bilateral thalami and basal ganglia (Figure 2). These ROIs were extracted directly from the JHU neonate atlas. Then, the mean value of these regions' CBF can be calculated and compared between groups.

In addition, structural images were evaluated for congenital and acquired lesions by two experienced neuroradiologists blinded to the patients' clinical history. Acquired abnormalities were divided into three categories (McQuillen et al., 2007; Dimitropoulos et al., 2013): (1) white matter injury (WMI) was characterized by focal/multifocal T1 hyperintensity in white matter, whether diffusion-weighted imaging displayed restricted or not; WMI was divided into three degrees (McQuillen et al., 2006): mild (≤ 3 foci and each ≤ 2 mm), moderate (>3 foci or any >2 mm), severe (>5% of total white matter volume). (2) Stroke was defined as a focal lesion with restricted diffusion related to an arterial territory; (3) intraventricular hemorrhage (IVH) was diagnosed by T1 hyperintensity on 3D T1 BRAVO and/or low signal on susceptibility-weighted angiography.

### Clinical variables

Clinical data of all subjects were collected and summarized by a doctor from the department of cardiovascular and thoracic surgery blinded to all neuroimaging findings, including sex, birth weight, gestational age (GA) at birth, postmenstrual age at the time of the scan, delivery way, head circumference at MRI. CHD cohort was further divided into cyanotic group and acyanotic group. The fundamental difference between cyanotic and acyanotic CHD is that the former has oxygenated and deoxygenated blood mixed together, while the latter does not.

### Statistical analyses

Statistical analyses were performed using SPSS v22.0 (IBM Corp., Armonk, NY, USA). The regional CBF values were regressed against GA and Cesarean delivery. After checking that the data conformed to the normal distribution, the regional CBF values were compared between the CHD and controls with an unpaired Student *t*-test and multiple comparison FDR correction. One-way analysis of variance (ANOVA) was used to compare ROI (right thalamus, left thalamus, right BG, and left BG) CBF between CHD subgroups (cyanotic CHD and acyanotic CHD) and controls, followed by *post-hoc* intergroup comparisons (Bonferroni correction). *p* < 0.05 was set as the significance threshold.

## Results

A total of 23 patients whose ASL images with unreliable quality such as motion artifacts and incomplete sequences were excluded. Ultimately, 39 subjects (22 CHD newborns, 17 healthy controls) fulfilled the inclusion criteria. Table 1 described subject demographics and delivery characteristics. The percentage of cesarean delivery for the CHD cohort (68%) exceeded that of controls (35%, *p* = 0.04). The average head circumference was obviously smaller than the controls (*p*< 0.001). Table 2 describes cardiac diagnoses. We categorized the CHD cases into acyanotic (10/22, 45.5%) vs. cyanotic (12/22, 54.5%) CHD.

**Table 1:** Demographic and characteristics of the neonates.

**Variable**	**CHD** **(Mean ±SD)**	**Controls** **(Mean ±SD)**	***p* value**
Male sex	36% (8/22)	41% (7/17)	0.77
Cesarean delivery	68% (15/22)	35% (6/17)	0.04
GA at birth (weeks)	38.2 ± 1.1	39.1 ± 1.0	0.02
Age at MRI (days)	7.6 ± 8.3	11 ± 7.1	0.29
GA at MRI (weeks)	39.3 ± 1.3	40.8 ± 1.6	0.19
Birth weight (kg)	3.2 ± 0.5	3.3 ± 0.4	0.26
Weight at MRI (kg)	3.3 ± 0.5	3.5 ± 0.5	0.07
Head circumference at MRI (cm)	33.6 ± 0.22	35.2 ± 0.35	<0.001

**Table 2:** Primary CHD diagnoses of the cohort.

**Acyanotic CHD**	**Number of subjects**	**Cyanotic CHD**	**Number of subjects**
Coarctation of the aorta	4	Transposition of the great arteries	3
Interruption of aortic arch	2	Total anomalous pulmonary venous connection	3
Ventricular septal defect	2	Pulmonary atresia with ventricular septal defect	2
Atrial septal defect with ventricular septal defect	1	Pulmonary atresia with intact ventricle septum	1
Pulmonary stenosis	1	Tetralogy of Fallot	1
		Single ventricle with pulmonary atresia	1
		Double outlet right ventricle	1
Total	10	Total	12

### Regional CBF of deep gray matter in CHD vs. controls

**Figure 3:**
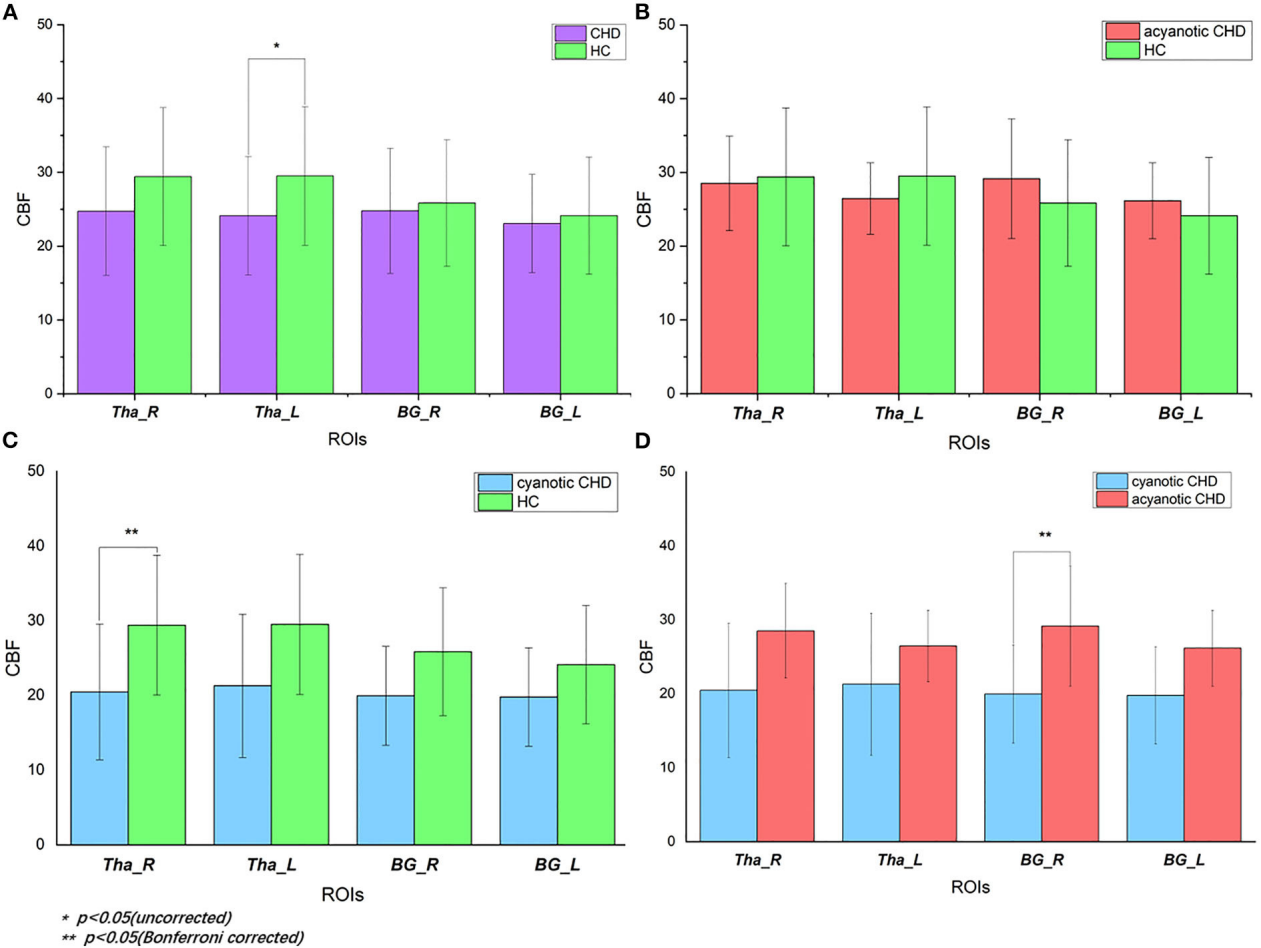
Regional CBF of deep gray matter in CHD and control subjects: **(A)** CBF values in CHD neonates vs. controls. When CHD group was assessed as a whole, it showed decreased CBF of left thalamus (*p* = 0.049) compared with controls. After FDR correction, there was no statistical difference. **(B)** CBF values in acyanotic CHD vs. controls. There was no statistically significant difference; **(C)** CBF values in cyanotic vs. controls. There was decreased CBF in right thalamus (*p* = 0.036) in cyanotic CHD newborns when compared with controls; **(D)** CBF values in cyanotic vs. acyanotic CHD. It showed lower right BG CBF (*p* = 0.042) in the cyanotic newborns compared with acyanotic newborns. HC, healthy controls; CHD, congenital heart disease; CBF, cerebral blood perfusion; Tha_R, right thalamus; Tha_L, left thalamus; BG_R, right basal ganglia; BG_L, left basal ganglia.

GA (De Vis et al., 2013) and delivery way (Morel et al., 2014) may be confounding variables. Given that, the analyses related to CBF differences in our study were corrected for the two variables. Mean regional perfusion in the right thalamus, left thalamus, right basal ganglia and left basal ganglia for the controls was 29.40, 29.50, 25.85, and 24.12 ml/100 g/min, respectively vs. 24.74, 24.11, 24.78 and 23.06 ml/100 g/min, respectively, for CHD cohort as a whole. Only the perfusion of the left thalamus (F = 4.128, *p* = 0.049) was statistically different. The left thalamus perfusion in the overall CHD was lower than that in controls. Although there were no statistical differences in regional CBF in the right thalamus (F = 3.04, *p* = 0.09), right basal ganglia (F = 0.353, *p* = 0.556), and left basal ganglia (F = 0.353, *p* = 0.556) between the overall CHD group and controls, it should be noted that deep gray matter perfusion in CHD neonates was lower than that in the healthy controls (Table 3, Figure 3). After FDR correction, there were no statistical differences.

**Table 3:** Comparison of regional CBF by ASL between CHD and controls (mL/100 g/min).

**ROI**	**CHD**	**Controls**	***t***	***p***	***p* (FDR-corrected)**
Right thalami	24.74 ± 8.70	29.40 ± 9.35	−1.744	0.090	0.18
Left thalami	24.11 ± 8.03	29.50 ± 9.39	−2.032	0.049^*^	0.18
Right BG	24.78 ± 8.47	25.85 ± 8.57	−0.594	0.556	0.556
Left BG	23.06 ± 6.64	24.12 ± 7.93	−0.594	0.556	0.556

Data are presented as mean ± standard deviation (normal distributed).

* p < 0.05.

### Regional CBF of deep gray matter in CHD subgroups vs. controls

There were inter-group differences in regional perfusion of the right thalamus (F = 3.854, *p* = 0.030) and right basal ganglia (F = 3.549, *p* = 0.039) with ANOVA (Table 4). After *post hoc* analysis, we found regional perfusion was lower in the right thalamus (*p* = 0.036) in cyanotic CHD than in controls. Right BG perfusion (*p* = 0.042) decreased in cyanotic CHD compared with acyanotic CHD (Table 5). The left thalamus and left BG perfusion did not have statistical differences among the three groups. No differences in regional BG or thalamus CBF were found between acyanotic CHD and controls (Figure 3).

**Table 4:** Comparison of regional CBF by ASL between CHD subgroups and controls (mL/100 g/min).

**ROI**	**Acyanotic CHD**	**Cyanotic CHD**	**Controls**	**F**	***p***
Right thalami	28.52 ± 6.41	20.47 ± 9.08	29.40 ± 9.35	3.854	0.030^*^
Left thalami	26.47 ± 4.84	21.29 ± 9.60	29.50 ± 9.39	3.050	0.060
Right BG	29.16 ± 8.12	19.95 ± 6.62	25.85 ± 8.57	3.549	0.039^*^
Left BG	26.17 ± 5.14	19.79 ± 6.60	24.12 ± 7.93	2.362	0.109

Data are presented as mean ± standard deviation (normal distributed).

* p < 0.05.

**Table 5:** Subgroup comparison results in the *post-hoc* analysis (Bonferroni correction).

**ROI**	**P1**	**P2**	**P3**
Right thalami	0.036^*^	0.130	1.000
Left thalami	0.056	0.538	1.000
Right BG	0.204	0.042^*^	0.974
Left BG	0.351	0.133	1.000

P1, cyanotic CHD vs. controls; P2, cyanotic CHD vs. acyanotic CHD; P3, cyanotic CHD vs. controls.

* p < 0.05.

### Structural MRI data

In the CHD group, there were 18.2% (4/22) of subjects with peri-operative brain injury, with WMI being the most common type of injury, characterized by small hemorrhagic and/or watershed infarcts. There were 3 with moderate WMI. The remaining 1 had mild WMI and intraventricular/germinal matrix. All controls had structurally normal brain MRI. Structural MRI Data were summarized in Table 6.

**Table 6:** Structural MRI of CHD neonates.

**Structural MRI data**	**Cyanotic CHD**	**Acyanotic CHD**
White matter injury	1	3
Stroke	0	0
Intraventricular hemorrhage	0	1
Global hypoxic ischemic injury	0	0

## Discussion

This study highlights impaired deep gray matter perfusion in neonates with CHD quantified by ASL. Regional perfusion was lower in the right thalamus in cyanotic CHD compared with controls. Regional perfusion of right BG in the cyanotic group was lower than that in the acyanotic group. When the CHD group was assessed as a whole, it showed slightly decreased CBF of the left thalamus compared with controls.

In neonates with CHD, abnormal cardiac physiological structure leads to changes in hemodynamics and disturbances in CBF (Leonetti et al., 2019). It is thought that smaller regional or global brain volumes, delayed microstructural development, increased white matter lactate level, and ischemic brain injury in some types of CHD especially complex CHD are associated with decreased cerebral oxygenation and hypoperfusion (Snider, 1985; Sethi et al., 2013; Jain et al., 2014). There was little data on CBF of the CHD population especially newborns. It is important to choose a safe and effective measurement method. Although color doppler imaging and near-infrared spectroscopy are noninvasive but they cannot quantify CBF values (Marin and Moore, 2011).

As a quantitative method, ASL is highly suitable and reliable to quantify and evaluate global and regional CBF in newborns and children (Wang et al., 2008; Jain et al., 2012). This is one of the few studies to evaluate ASL cerebral perfusion in CHD neonates. Licht et al. (2004) focused on global CBF in 25 neonates with severe CHD without subgroups and healthy controls. The other study (Nagaraj et al., 2015) reported regional BG and/or thalamus hypoperfusion in 43 neonates with diverse types of CHD compared with 58 healthy controls. Their ROIs were created by manually drawing 2 round 4 mm diameter spheres and the CBF was calculated as an average of both brain hemispheres. And we used a different method, ROI-based analysis, by registering corresponding CBF maps into the same Atlas space. Our study demonstrated similar results. The mechanism has not yet been fully clarified.

Several important changes occur during the transition from the fetus to post birth, with the pulmonary vascular resistance decreasing sharply and the systemic vascular resistance increasing. In normal neonates, these changes are accompanied by the closure of the ductus arteriosus, allowing for normal postnatal circulation (Peyvandi and Donofrio, 2018; Leonetti et al., 2019). For newborns with congenital heart disease, in this transitional period, it is challenging to maintain cerebral blood flow in the face of reduced pulmonary vascular resistance and diverse hemodynamic patterns such as common patent ductus arteriosus.

On the other hand, studies have shown delayed neurodevelopment in CHD neonates and increased preoperative brain injuries, similar to preterm infants (McQuillen et al., 2007; Miller et al., 2007). This delay in brain maturation that begins in the fetus, coupled with reduced postnatal pulmonary vascular resistance, impaired oxygen delivery, and increased postnatal brain metabolic demands may overwhelm the brain's autoregulation capacity (Peyvandi and Donofrio, 2018).

In healthy neonates, regional CBF in the deep gray structures is relatively higher for high metabolic demands (Huang and Castillo, 2008). As an important nuclei in the brain, impaired regional CBF in these areas may suggest decompensation or failure of the auto-regulatory mechanism of CBF, as demonstrated in CHD fetuses (Arduini et al., 2011).

Depending on the specific subtype of congenital heart disease, these abnormalities may vary and become more pronounced with more severe defects (Leonetti et al., 2019). For example, in infants with HLHS and aortic atresia, complex and abnormal heart structure lead to retrograde flow in the ascending aorta and decreases in blood flow to the brain. Another example is that in D-TGA, the low arterial oxygenated environment may affect the brain, thereby affecting the cerebral blood flow (Peyvandi and Donofrio, 2018; Leonetti et al., 2019). At present, the few studies are all single-center studies and necessarily include various forms of CHD due to sample size limitations. Further investigations using standardized techniques across large multi-center cohorts to allow subgroup analyses are critical to integrating cerebral findings with cardiac anatomy and establishing the pathophysiologic processes.

Our results also demonstrated a high prevalence of preoperative brain injury. The most common type of injury was WMI, observed in 13.6% of the study group, which was lower than some studies (Dimitropoulos et al., 2013). Probably it was caused by a small sample size. In addition, we found the CHD group showed a smaller average head circumference than healthy controls, as some previous studies did. This may be explained by delayed brain development and possible adverse neurodevelopmental outcome (Ortinau et al., 2012).

There are some limitations to the present study: firstly, small sample size precluded a more robust statistical analysis; second, we did not subgroup more specifically based on types of cardiac defect. This awaits more further data collection; third, we did not detect a correlation of CBF findings to neurodevelopmental outcomes; fourth, we didn't further segment the thalamus and investigate the different perfusion areas in thalami, especially in the myelinated nuclei. We will take this into the future study.

In conclusion, regional CBF of deep gray matter is disturbed in CHD neonates preoperatively. Cardiac physiology plays a crucial role in this. This study suggests the usefulness of ASL perfusion as a noninvasive method for assessing cerebral perfusion in CHD neonates and early intervention in the high-risk group to prevent the impairment of hypoperfusion.

## Data availability statement

The raw data supporting the conclusions of this article will be made available by the authors, without undue reservation.

## Ethics statement

The studies involving human participants were reviewed and approved by Ethics Committee, Shanghai Children's Medical Center, School of Medicine, Shanghai Jiao Tong University. Written informed consent to participate in this study was provided by the participants' legal guardian/next of kin. Written informed consent was obtained from the individual(s), and minor(s)' legal guardian/next of kin, for the publication of any potentially identifiable images or data included in this article.

## Author contributions

YS and YZ designed the experiment. YS, YZ, and YL collected the data. YS wrote the manuscript. WY analyzed the MRI data and prepared the figures. All authors contributed to the article and approved the submitted version.

## Funding

This study was supported by grants from the National Key Clinical Specialties Construction Program, National Natural Science Foundation of China (No.82171902), the Shanghai Committee of Science and Technology (No.17411965400).

## Conflict of interest

The authors declare that the research was conducted in the absence of any commercial or financial relationships that could be construed as a potential conflict of interest.

## Publisher's note

All claims expressed in this article are solely those of the authors and do not necessarily represent those of their affiliated organizations, or those of the publisher, the editors and the reviewers. Any product that may be evaluated in this article, or claim that may be made by its manufacturer, is not guaranteed or endorsed by the publisher.
